# Case report: Fatal lung hyperinflammation in a preterm newborn with SARS-CoV-2 infection

**DOI:** 10.3389/fped.2023.1144230

**Published:** 2023-05-23

**Authors:** Daniela Aguilar-Caballero, Jose M. C. Capcha, Veronica Caballero, Karen C. Young, Shahnaz Duara, Michael Borchetta, Ivan Gonzalez, Ali G. Saad, Keith A. Webster, Lina A. Shehadeh, Emmalee S. Bandstra, Augusto F. Schmidt

**Affiliations:** ^1^ Division of Neonatology, Department of Pediatrics, University of Miami Miller School of Medicine, Holz Children's Hospital/Jackson Memorial Hospital, Miami, FL, USA; ^2^ Division of Cardiology, Department of Medicine, University of Miami Miller School of Medicine, Miami, FL, USA; ^3^ Interdisciplinary Stem Cell Institute, University of Miami Miller School of Medicine, Miami, FL, USA; ^4^ Division of Pediatric Infectious Diseases, Department of Pediatrics, University of Miami Miller School of Medicine, Miami, FL, USA; ^5^ Division of Anatomic Pathology, Department of Pathology, University of Miami Miller School of Medicine, Miami, FL, USA; ^6^ Integene International, LLC, Miami, FL, United States; ^7^ Baylor College of Medicine, Everglades Biopharma, Cullen Eye Institute, Houston, TX, United States

**Keywords:** SARS-coV-2, newborn, hyperinflammation, lung, COVID-19, LDLR, heart

## Abstract

Vertical transmission of SARS-CoV-2 from mother to fetus is widely accepted. Whereas most infected neonates present with mild symptoms or are asymptomatic, respiratory distress syndrome (RDS) and abnormal lung images are significantly more frequent in COVID-19 positive neonates than in non-infected newborns. Fatality is rare and discordant meta-analyses of case reports and series relating perinatal maternal COVID-19 status to neonatal disease severity complicate their extrapolation as prognostic indicators. A larger database of detailed case reports from more extreme cases will be required to establish therapeutic guidelines and allow informed decision making. Here we report an unusual case of a 28 weeks' gestation infant with perinatally acquired SARS-CoV-2, who developed severe protracted respiratory failure. Despite intensive care from birth with first line anti-viral and anti-inflammatory therapy, respiratory failure persisted, and death ensued at 5 months. Lung histopathology showed severe diffuse bronchopneumonia, and heart and lung immunohistochemistry confirmed macrophage infiltration, platelet activation and neutrophil extracellular trap formation consistent with late multisystem inflammation. To our knowledge, this is the first report of SARS CoV-2 pulmonary hyperinflammation in a preterm newborn with fatal outcome.

## Introduction

Severe acute respiratory syndrome coronavirus 2 (SARS-CoV-2), the cause of COVID-19 disease, has broad clinical manifestations in adults that range from mild respiratory symptoms to acute respiratory failure. Maternal COVID-19 infection is associated with increased maternal death and serious morbidity (1, 2). Rates of all mode transmission of SARS-CoV-2 from COVID-19 positive mothers to neonates in different studies varies between 2 and 8% with some reports of vertical transmission as high as 5.3%, and COVID-19 related fatalities 0.08–0.35% (3–9). While most SARS-CoV-2 infected neonates are asymptomatic or mildly symptomatic, respiratory distress syndrome (RDS), elevated inflammatory markers and abnormal lung images are relatively common in symptomatic COVID positive newborns (2, 10–17). Vertical transmission via placental infection has been linked to maternal-fetal malperfusion and fetal hypoxia with consequences for possible negative outcomes (6, 10, 11, 18–23).

In adult patients, hyperinflammation contributes to the severity and mortality of COVID-19. Hyperinflammation is characterized by NF-kB activation and elevated systemic inflammatory markers, including cytokines IL-1β, IL-6, IL-8, IL-10, and TNF-α, mononuclear infiltrate with dysregulated macrophage activation and neutrophil activation with formation of neutrophil extracellular traps (NETs) that lead to further lung injury and microthrombi (15). Early complacency over the apparent resilience of COVID-19 infected children has been offset by the appearance of severe and in rare cases fatal disease associated with SARS-CoV-2 infection and multisystem inflammatory syndrome (MIS) of children (MIS-C) and neonates (MIS-N), and pediatric recurrence of a long-COVID-19 like condition involving earlier SARS-CoV-2 infection (PIMS-TS) (24–28). Such COVID-19 MIS differs from the cytokine storm of severe acute COVID-19, shares similarities with Kawasaki disease, and involves pathogenic autoimmune responses (29). Systematic literature reviews report more severe illness of such COVID-19 infected neonates relative to older children (9, 30, 31).

Roles for maternal age and COVID-19 severity in disease presentation and outcome of SARS-CoV-2 infected neonates is hotly disputed (1, 3, 32, 33). Discordance is exemplified by 3 recent reviews that found a lack of “concrete” evidence for vertical transmission or adverse effects of SARS-CoV-2 infection on pregnancy or newborns (32, 34, 35). Such discordance may be explained at least in part by an overriding majority of mildly symptomatic cases that differ only marginally from common complications of non-COVID pregnancies, and cases that were readily resolved by intensive care and anti-inflammation and/or antiviral therapy. Fatalities are often recorded only as statistics without cause of death, treatment, or disease etiology. Additional detailed case reports especially documenting extreme outcomes and pharmacological interventions are needed to identify causal links and determine treatment options. Here we report the case of a premature infant born at 28 weeks' gestation with SARS-CoV-2 infection that developed severe and protracted respiratory failure starting in the first week of life until death at 5 months with histopathological features of COVID-19 related pulmonary hyperinflammation. Immunohistology of autopsy specimens suggest late transition to multiorgan failure.

## Case report

A premature male was born at 28 weeks and five days gestation with a birth weight of 1,200 grams due to preterm labor. This was a dichorionic-diamniotic twin pregnancy. The co-twin was selectively terminated at an outside hospital at 21 weeks' gestation for multiple congenital anomalies secondary to amniotic band syndrome. The mother, a 23-year-old Hispanic with no reported history of serious disease tested positive for SARS-CoV-2 by polymerase chain reaction (PCR) tests 13 days before delivery and upon admission to the hospital. The mother was asymptomatic at presentation and reported no previous symptoms of COVID-19. She received antenatal steroids 29 days prior to delivery after she presented to the hospital with suspected rupture of membranes. Placental pathology of the co-twin showed acute chorioamnionitis with areas of fibrin deposition characteristic of intra-amniotic infection of an unidentified source.

**Figure 1:**
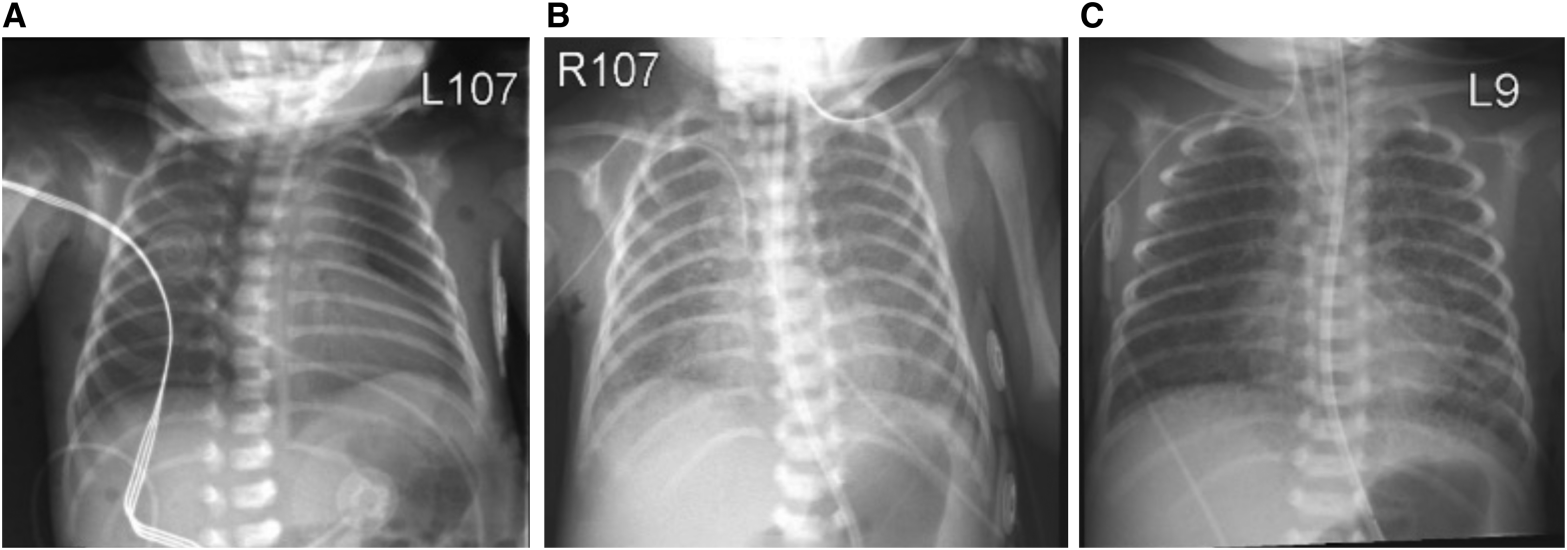
Chest x-ray at day of birth (**A**) showing mild diffuse granular opacification with progression of lung disease on day of life 5 (**B**) and 10 (**C**).

APGAR scores were 6 at 1 min and 9 at 5 min of life and initial resuscitation included positive pressure ventilation for one minute, followed by continuous positive airway pressure (CPAP) of 5cmH_2_O. The infant was admitted to the neonatal intensive care unit (NICU) on CPAP and FiO_2_ 0.21. Chest x-ray showed mild diffuse hazy granular opacities consistent with respiratory distress syndrome (Figure 1A). A nasopharyngeal SARS-CoV-2 PCR at 24 h postnatal age was positive and remained positive until day of life (DOL) 26. Inflammatory markers showed elevation of C-reactive protein (CRP) to 3 mg/dl (normal is <1 mg/dl) and interleukin-6 (IL-6) to 36.2 pg/ml (10x normal), which normalized by DOL 7. Serum antibodies (IgG, IgM) for SARS CoV-2 were undetectable on DOL 4, but present on DOL 19.

On DOL 5, increasing oxygen requirement (FiO_2_ 0.5) prompted treatment with dexamethasone (0.1 mg/kg every 12 h for 3 days, 0.05 mg/kg every 12 h for 2 days) and remdesivir (5 mg/kg loading dose, followed by 2.5 mg/kg/dose daily for 4 days). Chest x-ray at this point showed diffuse bilateral hazy opacities with increased perihilar markings (Figure 1B). Echocardiography was normal. By DOL 10, progressive respiratory failure required intubation, mechanical ventilation and FiO_2_ 1.00. Chest x-ray now showed bilateral interstitial infiltrates (Figure 1C). Due to deteriorating respiratory status, the dexamethasone dose was increased to 0.1 mg/kg every 6 h for 2 days with subsequent tapering. Anakinra (a synthetic IL-1 receptor antagonist) was started on DOL 11 at 1 mg/kg/dose every 12 h. FiO_2_ requirements improved to 0.6, whereupon dexamethasone and anakinra were discontinued by DOL 30.

On DOL 25, antibiotics were started for ventilator-associated pneumonia, presumedly due to respiratory cultures positive for Klebsiella pneumoniae and methicillin-resistant Staphylococcus aureus. On DOL 40, another course of dexamethasone was started at a dose of 0.15 mg/kg/day for 10 days, with some improvement in oxygen requirement, which worsened with dexamethasone taper. His FiO_2_ requirements increased further to 0.80–1.00, and a tracheostomy was performed on DOL 104. The infant continued to have severe respiratory failure requiring mechanical ventilation and high oxygen requirements despite multiple courses of empiric antibiotic treatment for presumed pneumonia. Echoencephalography was normal on DOL 5, 7, 14, 40, and 124. At 5 months of age, the infant's respiratory status progressively deteriorated and ventilatory support was withdrawn because of the extremely poor prognosis, and the infant soon expired.

**Figure 2:**
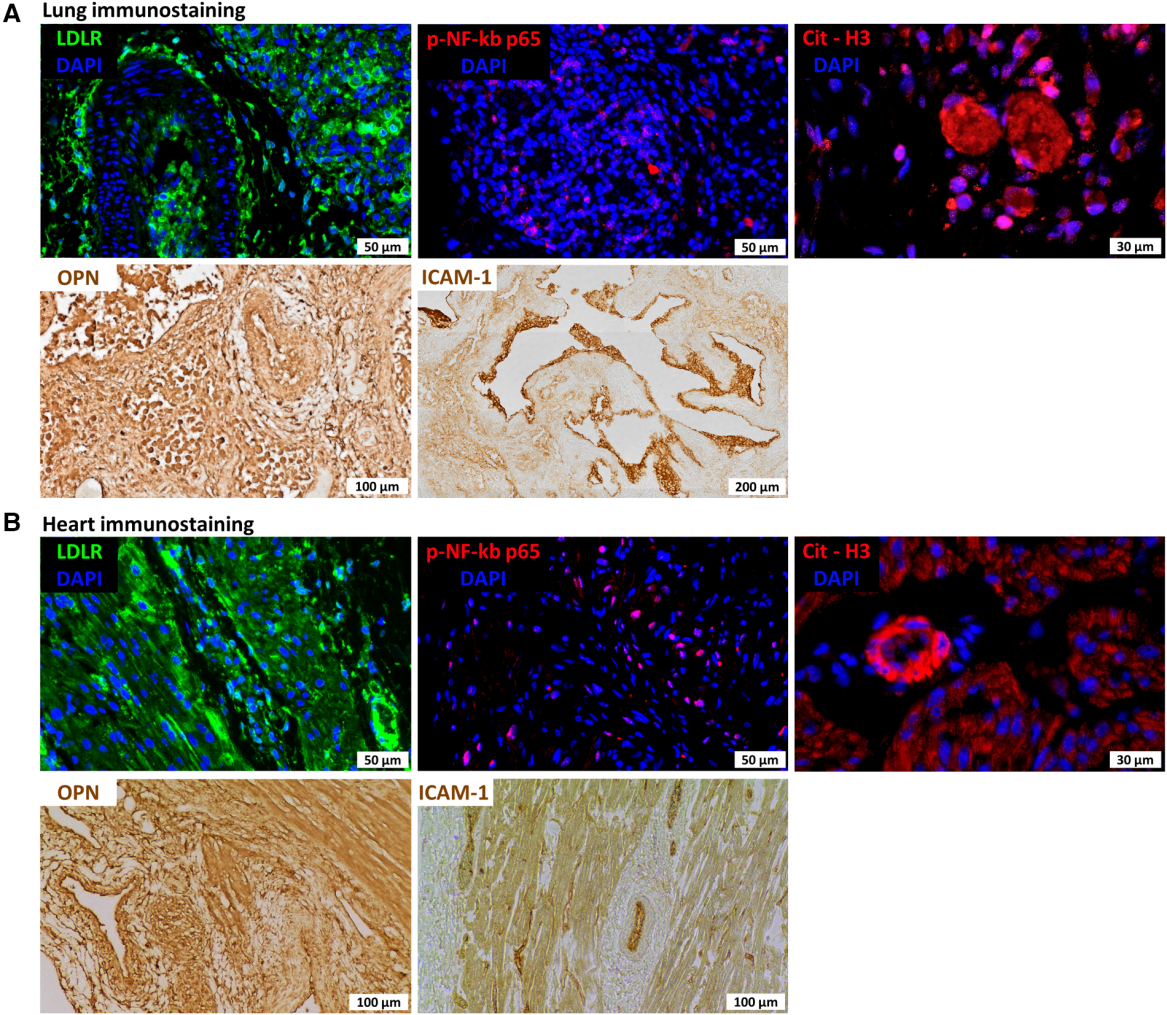
Immunostaining for lung (**A**) and heart (**B**) sections for low-density lipoprotein receptor (LDLR), nuclear factor kappa beta (NF-kb), citrullinated histone H3 (citH3), osteopontin (OPN), and intercellular adhesion molecule 1 (ICAM1).

Lung pathology showed severe bronchopneumonia. To investigate the plausible role of hyperinflammation in the etiology of the severe progressive respiratory failure and potential systemic effects in this infant, we performed additional stains in the lung and heart for proteins potentially involved in macrophage and platelet activation and neutrophil extracellular trap formation indicative of SARS CoV-2 hyperinflammation. We found, specifically, increased low-density lipoprotein receptor (LDLR) and phospo-NF-kB-p65 expression in lung macrophages and cardiomyocytes, increased osteopontin (OPN) and intracellular adhesion molecule 1 (ICAM-1) in blood vessels in the lung and heart (Figure 2), and increased citrullinated histone H3 (citH3) in the lung and blood vessels in the heart (Figure 2), indicating hyperinflammation with formation of NETs.

## Discussion

**Figure 3:**
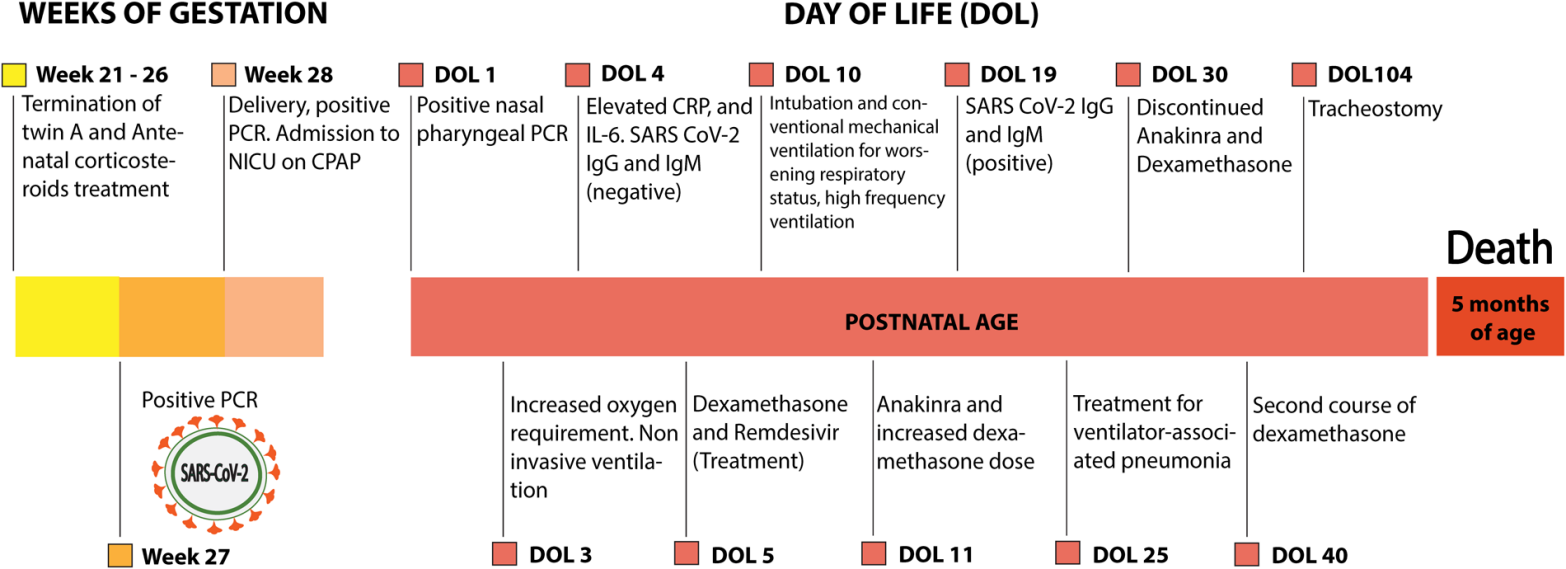
Outline of the case. CRP, C-reactive protein; CPAP, continuous positive airway pressure; IL-6, interleukin 6; NICU, neonatal intensive care unit; PCR, polymerase chain reaction.

In this case study, vertical transmission of SARS CoV-2 is indicated by the presence of maternal infection during the third trimester and at birth, positive nasopharyngeal tests of the neonate at 24 h and 26 days following immediate separation from the mother to an NICU. The case timeline is outlined in Figure 3. Abnormal chest x-rays, RDS, and elevated inflammatory markers at birth are also consistent with intrauterine transmission (7, 12, 36), and the presence of anti-SARS CoV-2 serum antibodies on DOL 19, but not DOL 4, is consistent with active neonatal innate and adaptive immune responses.

Our first line use of dexamethasone, remdesivir, and anakinra is supported by previous successes in hospitalized COVID-19 positive adults (37, 38), preterm infants with bronchopulmonary dysplasia (39), and severe COVID-19 neonates (40). Anakinra, a recombinant IL-1 receptor antagonist improved outcome in adult and pediatric patients with severe COVID-19 including suspected MIS-N and PIMS-TS cases. Despite the regimen of care that included empiric antibiotic treatment for presumed pneumonia, respiratory failure persisted and is assumed to be the cause of death at 5 months. Heart and lung inflammation, revealed at autopsy, suggest late progression to multisystem inflammatory syndrome with possible parallels to MIS-N and PIMS-TS (25–28, 41). The extreme outcome is consistent with previous reports on the absence of causal relationships between maternal age, COVID-19 severity, or disease history on SARS-CoV-2 vertically transmitted to the neonate. In this case the mother was 23 years of age without COVID symptoms or disease history. By comparison, two diabetic mothers aged 40 and 41 years with severe third trimester COVID-19 symptoms gave emergency C-section preterm births to vertically infected COVID-19 positive newborns that were discharged within 1-week of birth with treatments limited to antibiotics and/or standard care in the NICU (42, 43).

Elevated inflammation markers at birth and chest x-rays depicting progressive bilateral interstitial infiltrates paralleled by the appearance of anti-SARS CoV-2 antibodies and exacerbated RDS are consistent with prenatal exposure to inflammation (44), ongoing infection, and aggressive pulmonary inflammation. Because most mild to moderate, and even some severe cases of SARS CoV-2 related RDS are resolved by corticosteroids and/or anti-viral drugs, it seems possible that our subject did not respond to pharmacology because significant COVID-19 related fetal and neonatal injuries at an early stage provoked extremely imbalanced immune and inflammatory responses (45–47). COVID-19 immunopathogenesis involves two stages of progression: first, target-cell injury caused by aberrant immune reactions against substances from infected cells (PAMPs), and second, more extensive and widespread damage caused by systemic release of toxic substances from target-cell injury (DAMPs) (48, 49). In the case of pneumonia, the initial target cells are lower respiratory tract cells and the main etiologic agents are pathogenic substances from lesions in the lungs caused by infiltrating T cells in association with peripheral lymphopenia. Severe pneumonia is characterized by excess proinflammatory cytokines and proteolytic enzymes generated in phase I that cause major lung injury and an exaggerated phase II with more widespread inflammation (15). Neonates have robust innate but immature adaptive immune systems and may be protected against extreme COVID-19 related pneumonia because of muted phase II and suppressed hyperinflammation (37, 46). In our subject, severe bronchopneumonia evolved more than 4 months after COVID-19 infection along with apparent cardiovascular inflammation. Such etiology is consistent with prenatal infection and inflammation followed by major damage from a robust phase I innate responses and an overly aggressive phase II adaptive response that precipitated multisystem hyperinflammation with parallels to MIS-C and PIMS-TS (15, 16, 25–28, 41, 50).

This is the first report to describe SARS CoV-2 pulmonary hyperinflammation in a preterm newborn with fatal outcome. Lung pathology revealed severe bronchopneumonia with obliteration of airspaces by inflammatory cells without evidence of active infection at death. Immunohistochemical analysis of the infant's lungs and heart revealed diffuse expression of LDLR, NF-kb, and OPN, suggesting hyperinflammation. Furthermore, expression of ICAM-1 suggested vascular dysfunction (Figure 2), and citrullinated histone H3 (citH3) indicated formation of neutrophil extracellular traps (NETs). LDLR has been reported to bind with high affinity to SARS-CoV-2 spike protein (51). NF-kb is a master regulator of the immune function and is antagonized by dexamethasone, which has been demonstrated to improve outcomes in severe COVID-19 cases (52). Both circulating ICAM1 and OPN have been correlated with COVID-19 severity (53, 54). Furthermore, immunohistochemical analysis showing diffuse expression of citrullinated histone H3 (citH3) suggests formation of NETs which may be responsible for progressive respiratory failure and inability of the preterm lung to repair from the initial infection. In adult patients with COVID-19, the systemic presence of NET factors is associated with poor outcomes, and lung pathology has also confirmed the presence of microthrombi and NETs, suggesting that NETs may contribute to lung injury and microvascular disease in COVID-19 infection (55).

The unusual etiology of our case does not conform precisely with either MIS-N or PIMS-TS where intravenous dexamethasone with or without IgG are indicated (56, 57). Whereas RDS and lung inflammation were evident at birth consistent with third trimester maternal infection, normal echocardiography and echoencephalography scans and inflammatory markers that normalized during the first week suggested absence of systemic inflammation and unlikely MIS-N diagnosis (24). Serum anti-SARS CoV-2 antibodies suggest ongoing active infection at DOL 19. The infant remained under intensive care with optimal therapy while respiratory functions deteriorated until death at 5 months with hyperinflammation and probable multiorgan failure. PIMS-TS diagnoses following fetal or neonatal infections typically involve a latent period between infection and fulminant disease. For example, a case report of early trimester SARS-CoV-2 exposure of a fetus that culminated in severe, advanced COVID-19 disease in the newborn while the mother was already SARS-CoV-2 negative at delivery (58). In a second case, a 24-day neonate was rehospitalized with severe COVID-19 disease after normal presentation at birth. The mother had a history of SARS-CoV-2 infection but was also RT-PCR negative at delivery (28). In contrast, our case suggests prolonged dysregulation of innate immunity that culminated in hyperinflammation equivalent to that found in the lungs of adult COVID-19 patients. We postulate that these underlying features contributed to the severity of respiratory illness disproportionate to the prematurity and pathological picture of overwhelming bronchopneumonia.

## Data Availability

The original contributions presented in the study are included in the article, further inquiries can be directed to the corresponding author.
